# Genetic Diversity among Pseudorabies Viruses Isolated from Dogs in France from 2006 to 2018

**DOI:** 10.3390/pathogens8040266

**Published:** 2019-11-26

**Authors:** Céline Deblanc, Aurélie Oger, Gaëlle Simon, Marie-Frédérique Le Potier

**Affiliations:** ANSES, Ploufragan-Plouzané-Niort Laboratory, Swine Virology Immunology Unit, National Reference Laboratory and OIE Reference Laboratory for Aujeszky’s Disease, 22440 Ploufragan, France; aurelie.oger@anses.fr (A.O.); gaelle.simon@anses.fr (G.S.); marie-frederique.lepotier@anses.fr (M.-F.L.P.)

**Keywords:** pseudorabies, Aujeszky’s disease, phylogenetic analysis, gC encoding region, hunting dog, wild boar

## Abstract

Pseudorabies (PR), also known as Aujeszky’s disease, is an economically important disease for the pig industry. It has been eradicated in domestic pigs in many European countries, including France, but its causative agent—Suid Herpesvirus 1—is still circulating in wild boars. The risk of endemic PR in wild fauna lies in reintroducing the virus among domestic pigs and transmitting it to other mammals, especially hunting dogs for which the disease is rapidly fatal. As such infections are regularly reported in France, this study genetically characterized canine PR virus strains in the country to obtain information on their diversity and evolution. Partial sequencing of the glycoprotein C-encoding gene from 55 virus strains isolated from dogs between 2006 and 2018 showed that 14 strains belonged to genotype I-clade A and another 38 to genotype I-clade B, two clades usually reported in Western Europe. More surprisingly, three strains were found to belong to genotype II, suggesting an Asian origin. Genotype I-clade A strains exhibited the highest diversity as five geographically segregated genogroups were identified.

## 1. Introduction

Pseudorabies (PR), also known as Aujeszky’s disease, is a highly contagious viral disease affecting multiple animal species. Swine—including domestic pigs and wild boars—are the only natural hosts of the virus. The disease it causes is characterized by central nervous system disorders that lead to death in younger pigs, and by respiratory signs and reproductive disorders in adults, which can remain latently infected following clinical recovery [1]. In other mammals, such as carnivores and cattle, the infection rapidly induces a fatal neurological disease with clinical signs similar to those of rabies [2]. PR is caused by Suid Herpesvirus 1, a member of the *Herpesviridae* family. The PR virus (PRV) is an enveloped double-stranded DNA virus with a genome of 143 kb in length encoding for 70 different proteins [3]. Its UL44 gene encodes glycoprotein C (gC), which plays a role in virus attachment to cell receptors and is one of the major targets for the host’s immune system [2]. The partial sequencing of the gC-encoding gene, which is one of the most variable regions of the genome and has the largest number of reported sequences [4,5], showed that whatever their host species, PRV strains can be classified as genotype I or genotype II, mostly depending on their geographical location, i.e., respectively, Europe/America or Asia [6,7,8]. Within genotype I, PRV strains can be divided into two clades: clade A, which contains PRV strains from Europe and America, and clade B with strains only originating from Western Europe [9,10,11]. Besides this variability dependent on geographical location, it appears that strains may evolve differently according to their host (i.e., domestic swine or wild boars) [12]. Italian strains of clade A could be classified into three groups, for example: “Italian clade 1” containing all the strains isolated from wild boars and hunting dogs, and “Italian clade 2” and “Italian clade 3”, containing strains isolated from domestic pigs or epidemiologically-related dogs, the most recent strains belonging to clade 2 [13].

Due to the significant economic losses caused by PR in the pig industry, an eradication policy including the slaughter of infected livestock and/or preventive vaccination of pigs was implemented in France from 1990 to 2006. Although PRV remained in the domestic pig population of Corsica, a Mediterranean island with semi-extensive herds, the eradication program was successful in mainland France and resulted in its PR-free status in 2008 (Decisions 2008/185/EC [14] and 2008/269/EC [15]). However, serological surveys indicated that PRV was still circulating in wild boars, with prevalence levels reaching 20–50% in regions with the highest population density [16,17], especially in the north-east and center of mainland France, as well as in Corsica [17,18,19]. The risk of such endemic PR in wild boars lies in reintroducing PRV into pig herds, especially those on outdoor farms due to potential contact between domestic pigs and wild boars [20]. Indeed, since the end of the eradication program in the pig industry, three PR outbreaks were detected through serological analyses in open-air pig farms in 2010, 2018, and 2019 [21,22,23]. Moreover, infected wild boars can also be a source of contamination for other mammals. Several cases of PRV-infected dogs have thus been reported in France in recent years [24,25,26,27]. Most often, these dogs presented clinical signs of PR after wild boar hunting and died within a few days. In this study, the PRV strains isolated from dogs in France from November 2006 to June 2018 were partially sequenced in order to obtain information on the nature, evolution, and geographical distribution of the PRV strains circulating among wild boars, which constitute a PRV reservoir.

## 2. Results

### 2.1. Characterisation of French Canine PRV Strains

**Figure 1:**
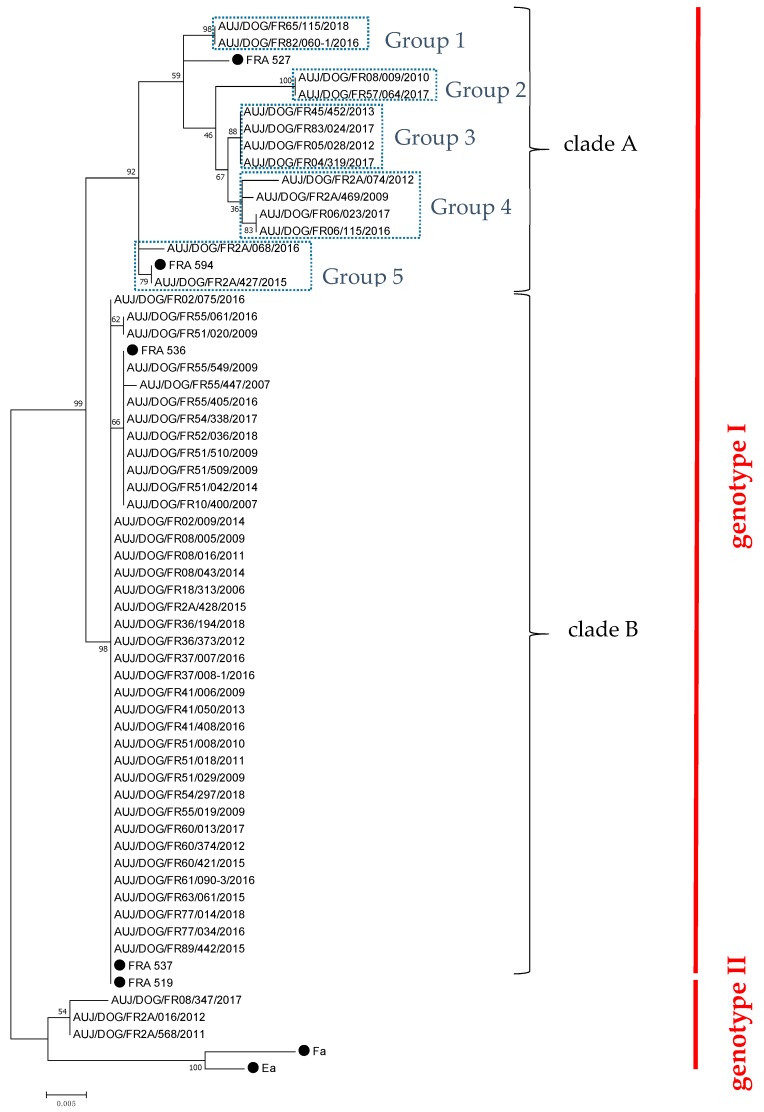
Phylogenetic tree of French canine PRV isolates based on partial nucleotide sequencing of the gC-encoding gene (UL44, 659 to 680 nt). The bootstrap percentage values are indicated at the nodes. The sequences of French PRV strains previously published [10], and sequences of two Asian PRV strains (Ea and Fa, GenBank accession numbers AF158090.1 and AF403051.1, respectively) were used to define the different genotypes and clades. These strains are indicated by a black dot.

Fifty-five PRV strains were propagated onto Porcine Kidney (PK-15) cells from 138 brains sampled from November 2006 to June 2018 (Table 1). Of these 55 strains, 32 were obtained from hunting dogs having been in contact with a wild boar, and two were from dogs that died after contact with a wild carnivore (respectively, a fox and a badger). No epidemiological information was available for the other 21 canine hosts. Partial UL44 gene sequences were obtained for each of these 55 PRV canine strains. Phylogenetic analyses showed that 52 (94.5%) of them belonged to PRV genotype I, including 14 strains (25.5% of total strains) isolated between 2009 and 2018 classified in clade A, and 38 strains (69% of total strains) obtained throughout the study period classified in clade B (Figure 1). Strains from genotype I-clade B appeared highly similar, indicating marked stability over the 12-year collection period, while those from clade A were more diverse, leading to five different genogroups within this clade (groups 1 to 5) (Figure 1). Apart from strains belonging to genotype I, the other three isolates (5.5% of strains) obtained in 2011, 2012, and 2017, respectively, belonged to PRV genotype II (Figure 1).

### 2.2. Geographical Distribution of PRV Strains Isolated from Dogs in France from 2006 to 2018

The PRV strains were isolated from dogs living in 25 French administrative *départements*.

**Figure 2:**
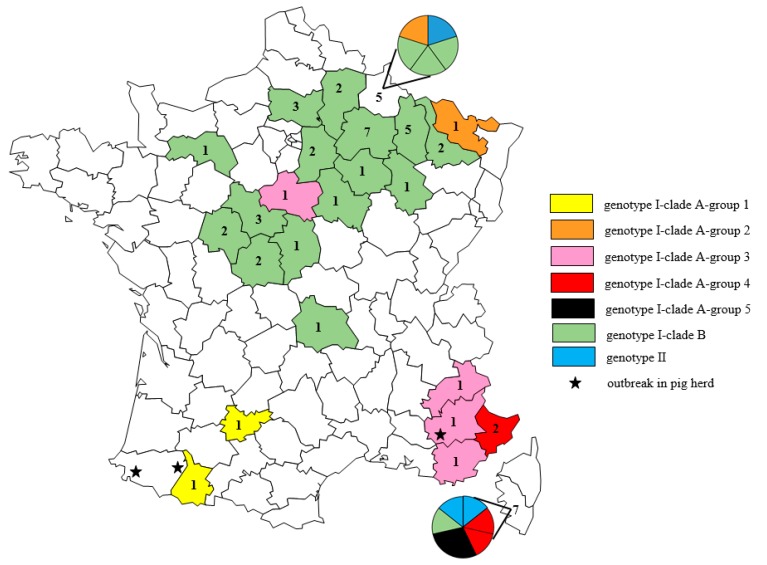
Geographical distribution of PRV strains isolated from dogs in France between November 2006 and June 2018. Black lines delineate administrative *départements*. The number of PRV strains isolated from dogs is shown in each colored *département*. The colors indicate the genotype, clade, or group that these strains belong to according to the legend provided. Index cases of outbreaks in domestic pig herds in 2010, 2018, and 2019 are indicated for information by a black star.

The majority (11/14) of strains from genotype I-clade A (I-A) were isolated in the southern half of the country (Figure 2). Interestingly, these I-A strains were geographically distributed according to their genogroup. Indeed, strains from Group 1 were identified in the south-western part of France, and those from Group 3 were mostly from the south-east, except one from the center. Strains from Group 4 were also found in a south-eastern *département* near Italy, as well as in Corsica. Strains from Group 5, however, were found only in Corsica (Figure 2). Finally, strains from Group 2 were only detected in the north-east, in two *départements* close to Belgium, Luxembourg, and Germany.

Unlike I-A strains, only two out of 38 strains from genotype I-clade B were isolated in the southern half of France (one in the center and one in Corsica (Figure 2)). Thus, 36/38 I-B strains were concentrated in the central and eastern part of the northern half of the country.

Two of the three genotype II strains were isolated in Corsica, but the third one was isolated quite recently in the Ardennes *département* in northern France, on the border with Belgium.

### 2.3. Comparison of French Canine PRV Strains with PRV Strains Isolated in Other Countries

**Figure 3:**
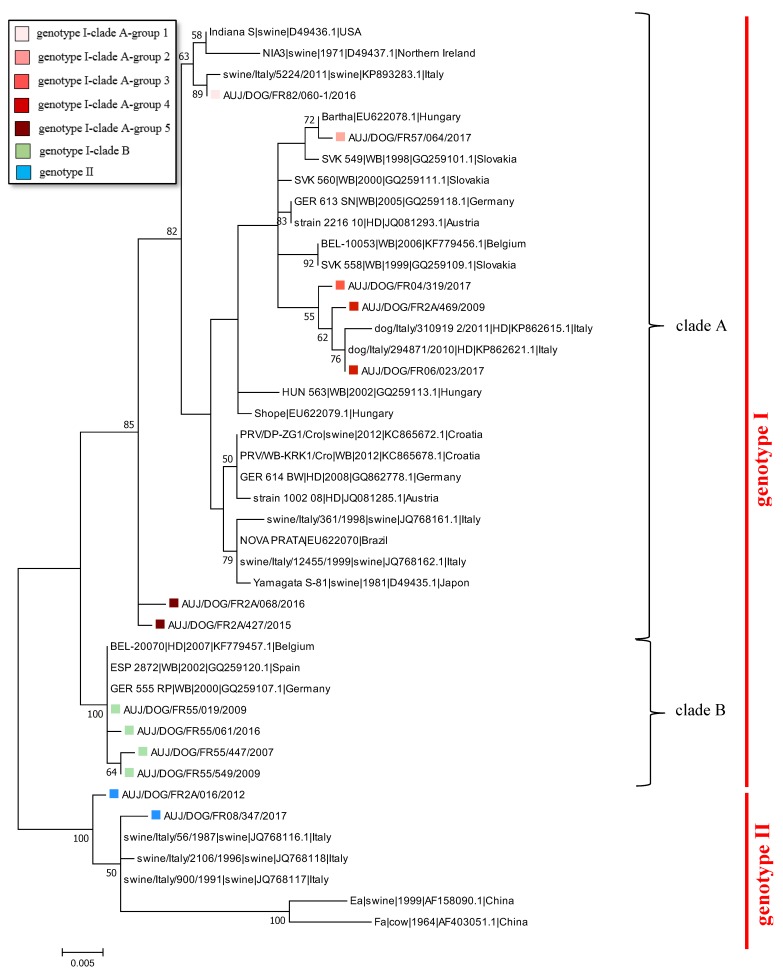
Phylogenetic tree based on the partial sequencing of the gC-encoding gene of PRV strains isolated in France and other countries. French sequences are identified by their name and indicated by a colored square. The colors indicate the genotype, clade, or group of these French strains according to the legend provided on the figure. Only a selection of French strains representative of each genogroup is indicated. The sequences of other strains are identified by their name/species/year of isolation/GenBank accession number/country (if known). Bootstrap values under 50 are not indicated. WB = wild boar, HD = hunting dog.

Phylogenetic analyses performed with partial sequences of the UL44 gene from PRV strains identified in other countries showed that French canine strains from genotype I-clade A-Group 1 clustered with Italian swine strains from “Italian clade 2” [13] (Figure 3). I-A Group 2 strains were found to be related to the attenuated strain Bartha used for vaccines [28] and other isolates obtained from wild boars in Slovakia. Strains from Groups 3 and 4 were linked to strains isolated from hunting dogs in Italy and belonging to “Italian clade 1” [13]. Finally, Group 5 strains did not cluster with any foreign PRV strains. French canine strains from genotype I-clade B grouped with PRV strains isolated from wild boars or hunting dogs in Belgium, Germany, and Spain. Lastly, the French canine strains belonging to genotype II grouped with the other three genotype II strains isolated from Italian swine in 1987, 1991, and 1996 (Figure 3).

### 2.4. Analysis of the Translated Protein Sequences of French Canine PRV Strains

**Figure 4:**
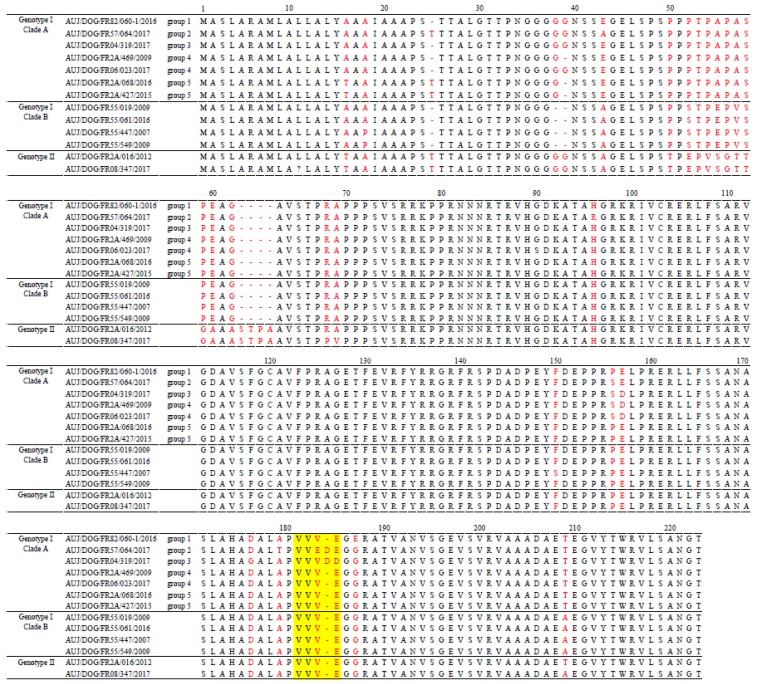
Alignment of the gC-deduced amino acid sequences from a selection of French canine PRV strains. Positions with deletions, insertions, or substitutions are in red. The hot spot region is indicated in yellow.

A comparison of partial gC protein sequences of French canine PRV strains showed that the I-A and I-B strains could be distinguished on the basis of amino acids in positions 38, 52, 55, 57, and 209 (Figure 4). Indeed, clade A strains were characterized by respectively G, P, A, A, and T at these positions, whereas clade B strains were characterized by a deletion at position 38 and S, E, V, and A at positions 52, 55, 57, and 209, respectively. Within clade A, strains from the five genogroups were differentiated by mutations or deletions of amino acids at positions 16, 25, 39, 43, 96, 156–157, and 176. Finally, genotype II strains had many mutations and insertions of amino acids between positions 50 and 69 compared to genotype I strains (Figure 4). At position 181–185, which corresponds to a hot spot region showing the largest variations of amino acids within this gC portion [28,29], the French canine PRV strains exhibited profiles VVVE (49/55 strains), VVVDD (4/55 strains) or VVEDE (2/55 strains) but not profile ALDDD that was reported to be characteristic of the domestic pig strains included in “Italian clade 3” [13].

## 3. Discussion

This study aimed to provide more comprehensive knowledge of PRV strains circulating in France and their distribution. All the PRV strains isolated at the French National Reference Laboratory from November 2006 to June 2018 were thus partially sequenced. They were all isolated from dogs that had died of PR, probably after contact with an infected animal, which in most cases, was during a wild boar hunt. Thus, these canine strains probably traced back to those circulating in wild boars. Phylogenetic analyses confirmed that strains belonging to both genotype I-clade A and genotype I-clade B were co-circulating in France without any particular distribution of genotypes according to years, in accordance with a previous study [10]. The latter did not, however, provide any information on geographical distribution, since only five French strains isolated from wild boars and dogs in the north-east were sequenced [10]. The present study identified clade A strains mostly in the southern part of the country, and clade B strains in the north-eastern and central regions. However, strains belonging to clade B have also been described in Spain, Belgium, and Germany [10,11], thus it is possible that clade B strains could also be present in south-western France, in *départements* close to the Spanish border, as very few samples were collected in this region. Although PR seroprevalence in wild boars was found to be lower in this region than in north-eastern or central France [17,18,19], there is still a risk of PRV transmission from wild boars to domestic pigs, as there are many outdoor pig herds in this area [19]. Indeed, two PR outbreaks occurred in outdoor pig herds in the Pyrénées-Atlantiques *département* in 2010 and 2018, respectively, but the strains involved could not be identified [21,22]. It is important, therefore, to obtain more data on the PRV strains circulating in the wild boar population in this region.

Strains belonging to clades I-A and I-B were previously reported to be co-circulating in three European countries—Belgium, Germany, and France [10,11]—but this study provides the first evidence of the circulation of genotype II strains in France. PRV strains belonging to genotype II have been commonly identified in Asia [7], but rarely reported in Europe. Three genotype II strains were detected 20 years ago on farms in Italy, where only I-A strains are usually detected [30]. These strains were probably introduced into Europe via the importation of live pigs from the Asian continent [12,30], but until our study, they had not been isolated in Europe since then. Based on our phylogenetic studies, these strains of Asian origin could have been transmitted from domestic pigs to wild boars, which served as a reservoir allowing PRV transmission to hunting dogs. A similar but reverse situation was observed on Chinese farms, where some PRV strains were found to belong to genotype I-clade A, although the vast majority belonged to genotype II [6].

It is well known that PRV strains can spill over from the wild boar population to the domestic pig population and vice versa. Such bidirectional transmissions have been experimentally demonstrated [31,32], and phylogenetic analyses have confirmed that strains related to wild boars can cluster with those related to domestic pigs [11,33,34]. This is indeed the case here, with two French strains isolated from hunting dogs belonging to I-A Group 1 clustering with strains isolated from domestic pigs in other countries. Other clusters, however, only contained viruses isolated from wildlife (for example, strains from clade B) or from domestic pigs (for example, “Italian clade 3” [13]), which suggests an adaptation of some PRV strains to their host. 

In the present study, all the strains complied with pattern VVVE, VVVDD, or VVEDE in the hot spot region at positions 181–185, which was also observed in other European wild boar strains [10]. None of them presented the ALDDD profile that was considered as characteristic of some PRV strains isolated from domestic pigs, notably those of “Italian clade 3” [13]. This putative swine-specific sequence was also present in French strain FR527 described previously [10]. Strain FR527 was isolated from a hunting dog in the north of France, but additional analysis of restriction fragment length polymorphisms (RFLP) of the entire genome revealed a pig-specific PRV profile, suggesting a recent introduction from domestic pigs into the wild boar population [10]. The complete change from VVVE to ALDDD was associated with changes in the hydrophobicity profile, which would cause the gC protein to be more antigenically exposed in this region [29]. Many other variations in amino acid residues were observed between the various PRV strains, as for example, at positions 16, 25, 38, or 39. Many of them have also been observed in previous studies conducted in other countries [10,13,35], but these differences in gC did not seem to relate to biological differences in vitro [12]. In vivo, it was demonstrated that two Belgian PRV strains differing from each other by the amino acid at position 18 presented attenuated clinical signs in adult pigs, but only one of them induced a major respiratory and neurological disease in young piglets [36]. Sequencing the partial UL44 gene, which is only a small part of the PRV genome, is not sufficient to assume a possible difference in the pathogenicity of PRV strains. To further characterize French canine PRV strains in terms of pathogenicity and to better evaluate their potential impact in the event of their introduction into a pig farm, it would be useful to further sequence other parts of the PRV genome, for example the genes encoding the gE and gI proteins that have been shown to contribute to the virulence of PRV strains in pigs [37]. An in silico analysis of virulence markers could be performed with these sequencing data to obtain information on the relevance of conducting experimental infection studies in pigs to compare the pathogenicity of the different genotypes of French canine PRV strains.

To conclude, this study evidenced the marked genetic diversity of canine PRV strains that are assumed to reflect those circulating in wild boars. However, PRV strains directly collected from hunted wild boars need to be sequenced to confirm this diversity. Faced with the large increase in wild boar densities for the last 30 years in France [38], and consequently, an increased risk of PRV transmission from wild to domestic animals, it appears important to continue PRV monitoring and investigations. This obviously requires the involvement of hunters and veterinarians to collect new canine and wild boar samples, notably in the areas under-represented in this study such as south-eastern *départements* around the Mediterranean Sea, which have a large wild boar population and for which we had no/few samples.

## 4. Material and Methods

### 4.1. PRV Detection and Isolation from Dog Samples

Requests for laboratory diagnosis of PR in sick non-Suidae animals such as dogs and cats were made by veterinarians on a voluntary basis since notification of the disease was not mandatory in these animals. From November 2006 to June 2018, brain samples from 138 animals presenting neurological symptoms characterized by severe pruritus with self-mutilation were first tested for rabies then, if negative, were sent to the National Reference Laboratory for PR in France for PRV detection.

From 2006 to 2009, 38 samples were received and tested for PRV infection by propagation onto Porcine Kidney (PK-15) cells following the standard procedure [39]. The 100 samples received between 2010 and 2018 were first tested by real-time qualitative PCR targeting the gD-encoding gene (ADIAVET PRV REALTIME kit, Bio-X Diagnostics, Rochefort, Belgium), a fully validated method for PRV genome detection [40]. An attempt was then made to isolate the virus from the 67 PCR-positive samples, as described above.

Each isolated PRV strain was identified as follows: AUJ/species/FR (for France), followed by an identification number for the area, i.e., French administrative *département* where the strain was isolated, sample identification number, and year of isolation.

### 4.2. Sequencing of the gC-Encoding Gene

The UL44 gene was partially sequenced at its 5′ end, in gC-encoding ORF, location 54029-55468 [3]. DNA was extracted from cell-propagated PRV strains using DNeasy Blood and Tissue Kit (Qiagen, Hilden, Germany), and then amplified with the Dream Taq DNA Polymerase (Thermo Fisher Scientific, Waltham, MA, USA) using the gC-specific primers, and PCR protocol previously described [4,28]. Amplification steps were performed in a GeneAmp PCR System 9700 Thermocycler (Applied Biosystems, Foster City, CA, USA). Amplification products were separated in a 1% agarose gel and the fragments of ~800 pb were purified using the NucleoSpin Gel and PCR Clean-up kit (Macherey Nagel, Düren, Germany) according to the manufacturer’s instructions. Purified PCR fragments were quantified with the Qubit dsDNA HS assay kit (Thermo Fisher Scientific, Waltham, MA, USA) and 15–20 ng were sequenced using the BigDye Terminator v3.1 Cycle Sequencing Kit (Thermo Fisher Scientific, Waltham, MA, USA) and the same primers as for initial amplification, as well as additional internal primers (5′-GATGCTCGCTCTGCTGGC-3′ and 5′-GCTCGTCAAAGTACTCGGGGT-3′). Sequencing was performed on the automatic DNA sequencer ABI 3130 Genetic Analyzer (Thermo Fisher Scientific, Waltham, MA, USA). The sequences were edited and assembled with the Vector NTI Advance 11.0 software (Life Technologies, Carlsbad, CA, USA) and submitted to GenBank. Their accession numbers are given in Table 1.

### 4.3. Phylogenetic Analysis

The UL44 gene partial sequences of the PRV canine strains isolated in France were aligned by ClustalW with MEGA7 software [41], and the alignment was optimized manually. The corresponding sequences (659 to 680 pb) of PRV strains isolated in France in previous studies or in other countries [9,10] were retrieved from GenBank and included in the phylogenetic analyses. Trees were generated with MEGA7 software using the maximum likelihood method and the Tamura 3-parameter substitution model (T92 G+I), which was chosen according to the Bayesian information criterion (BIC). Bootstrap values were derived from 1000 replicates.

The deduced protein sequences were obtained by translating the nucleotide sequences with MEGA7 software.

## Figures and Tables

**Table 1:** Pseudorabies virus (PRV) strains isolated in French dogs from November 2006 to June 2018.

Year	Isolate ID	Location (Name of French Administrative *Département*)	gC Genotype	Clade	GenBank Accession Number
2006	AUJ/DOG/FR18/313/2006	Cher	I	B	MN590185
2007	AUJ/DOG/FR10/400/2007	Aube	I	B	MN590184
	AUJ/DOG/FR55/447/2007	Meuse	I	B	MN590214
2009	AUJ/DOG/FR08/005/2009	Ardennes	I	B	MN590179
	AUJ/DOG/FR41/006/2009	Loir-et-Cher	I	B	MN590197
	AUJ/DOG/FR55/019/2009	Meuse	I	B	MN590211
	AUJ/DOG/FR51/020/2009	Marne	I	B	MN590203
	AUJ/DOG/FR51/029/2009	Marne	I	B	MN590204
	AUJ/DOG/FR2A/469/2009	Corse-du-Sud	I	A	MN590191
	AUJ/DOG/FR51/509/2009	Marne	I	B	MN590206
	AUJ/DOG/FR51/510/2009	Marne	I	B	MN590207
	AUJ/DOG/FR55/549/2009	Meuse	I	B	MN590215
2010	AUJ/DOG/FR51/008/2010	Marne	I	B	MN590201
	AUJ/DOG/FR08/009/2010 *	Ardennes	I	A	MN590180
2011	AUJ/DOG/FR08/016/2011	Ardennes	I	B	MN590181
	AUJ/DOG/FR51/018/2011	Marne	I	B	MN590202
	AUJ/DOG/FR2A/568/2011	Corse-du-Sud	II		MN590192
2012	AUJ/DOG/FR2A/016/2012	Corse-du-Sud	II		MN590186
	AUJ/DOG/FR05/028/2012	Hautes-Alpes	I	A	MN590176
	AUJ/DOG/FR2A/074/2012	Corse-du-Sud	I	A	MN590188
	AUJ/DOG/FR36/373/2012	Indre	I	B	MN590194
	AUJ/DOG/FR60/374/2012	Oise	I	B	MN590218
2013	AUJ/DOG/FR41/050/2013	Loir-et-Cher	I	B	MN590198
	AUJ/DOG/FR45/452/2013	Loiret	I	A	MN590200
2014	AUJ/DOG/FR02/009/2014	Aisne	I	B	MN590173
	AUJ/DOG/FR51/042/2014	Marne	I	B	MN590205
	AUJ/DOG/FR08/043/2014	Ardennes	I	B	MN590182
2015	AUJ/DOG/FR63/061/2015	Puy-de-Dôme	I	B	MN590221
	AUJ/DOG/FR60/421/2015	Oise	I	B	MN590219
	AUJ/DOG/FR2A/427/2015	Corse-du-Sud	I	A	MN590189
	AUJ/DOG/FR2A/428/2015	Corse-du-Sud	I	B	MN590190
	AUJ/DOG/FR89/442/2015	Yonne	I	B	MN590227
2016	AUJ/DOG/FR37/007/2016	Indre-et-Loire	I	B	MN590195
	AUJ/DOG/FR37/008-1/2016	Indre-et-Loire	I	B	MN590196
	AUJ/DOG/FR77/034/2016	Seine-et-Marne	I	B	MN590224
	AUJ/DOG/FR82/060-1/2016	Tarn-et-Garonne	I	A	MN590225
	AUJ/DOG/FR55/061/2016	Meuse	I	B	MN590212
	AUJ/DOG/FR2A/068/2016	Corse-du-Sud	I	A	MN590187
	AUJ/DOG/FR02/075/2016	Aisne	I	B	MN590174
	AUJ/DOG/FR61/090-3/2016	Orne	I	B	MN590220
	AUJ/DOG/FR06/115/2016	Alpes-Maritimes	I	A	MN590178
	AUJ/DOG/FR55/405/2016	Meuse	I	B	MN590213
	AUJ/DOG/FR41/408/2016	Loir-et-Cher	I	B	MN590199
2017	AUJ/DOG/FR60/013/2017	Oise	I	B	MN590217
	AUJ/DOG/FR06/023/2017	Alpes-Maritimes	I	A	MN590177
	AUJ/DOG/FR83/024/2017	Var	I	A	MN590226
	AUJ/DOG/FR57/064/2017	Moselle	I	A	MN590216
	AUJ/DOG/FR04/319/2017	Alpes-de-Haute-Provence	I	A	MN590175
	AUJ/DOG/FR54/338/2017	Meurthe-et-Moselle	I	B	MN590210
	AUJ/DOG/FR08/347/2017	Ardennes	II		MN590183
2018	AUJ/DOG/FR77/014/2018	Seine-et-Marne	I	B	MN590223
	AUJ/DOG/FR52/036/2018	Haute-Marne	I	B	MN590208
	AUJ/DOG/FR65/115/2018	Hautes-Pyrénées	I	A	MN590222
	AUJ/DOG/FR36/194/2018	Indre	I	B	MN590193
	AUJ/DOG/FR54/297/2018 *	Meurthe-et-Moselle	I	B	MN590209

* indicates that strains were isolated from dogs having been in contact with a fox or badger.
